# Pyrophosphate: fructose-6-phosphate 1-phosphotransferase (PFP) regulates carbon metabolism during grain filling in rice

**DOI:** 10.1007/s00299-016-1964-4

**Published:** 2016-03-18

**Authors:** Erchao Duan, Yihua Wang, Linglong Liu, Jianping Zhu, Mingsheng Zhong, Huan Zhang, Sanfeng Li, Baoxu Ding, Xin Zhang, Xiuping Guo, Ling Jiang, Jianmin Wan

**Affiliations:** National Key Laboratory for Crop Genetics and Germplasm Enhancement, Jiangsu Plant Gene Engineering Research Center, Nanjing Agricultural University, Nanjing, 210095 China; State Key Laboratory of Rice Biology, China National Rice Research Institute, Chinese Academy of Agricultural Sciences, Hangzhou, 310006 China; National Key Facility for Crop Gene Resources and Genetic Improvement, Institute of Crop Science, Chinese Academy of Agricultural Sciences, Beijing, 100081 China

**Keywords:** *Oryza sativa*, Floury endosperm, Pyrophosphate: fructose-6-phosphate 1 phosphotransferase (PFP), Carbon metabolism

## Abstract

*****Key message***:**

**Decreased PFPase activity in rice perturbs the equilibration of carbon metabolism during grain filling but has no visible phenotypic effects during the vegetative and reproductive growth stages.**

**Abstract:**

Starch is a primary energy reserve for various metabolic processes in plant. Despite much advance has been achieved in pathways involved in starch biosynthesis, information was still lacked for precise regulation related to carbon metabolism during seed filling in rice (*Oryza sativa*). The objective of this study was to identify and characterize new gene associated with carbon metabolism during grain filling. By screening our chemical mutant pool, two allelic mutants exhibiting floury endosperm were isolated. No visible phenotypic defects were observed during both the vegetative and reproductive growth stages, except for the floury-like endosperm of grains with significantly reduced kernel thickness, 1000-grain weight and total starch content. Map-based cloning revealed that the mutant phenotypes were controlled by a gene encoding pyrophosphate: fructose-6-phosphate 1-phosphotransferase (PFP, EC 2.7.1.90) β subunit (PFP_β_), which catalyzes reversible interconversion between fructose-6-phosphate and fructose-1, 6-bisphosphate. The identity of *PFP*_*β*_ was further confirmed by a genetic complementation test. Subcellular analysis demonstrated that PFPβ was localized in cytoplasm. Quantitative PCR and histochemical staining indicated *PFP*_*β*_ was ubiquitously expressed in various tissues. Furthermore, we found PFP_*β*_ could express in both the early and late phases of starch accumulation during grain filling and decreased activity of *PFP*_*β*_ in *pfp* mutants resulted in compromised carbon metabolism with increased soluble sugar contents and unfavorable starch biosynthesis. Our results highlight PFP_β_ functions in modulating carbon metabolism during grain filling stage.

**Electronic supplementary material:**

The online version of this article (doi:10.1007/s00299-016-1964-4) contains supplementary material, which is available to authorized users.

## Introduction

Starch is the primary storage polysaccharide in many sink tissues in plants, such as potato (*Solanum tuberosum*) tubers and rice (*Oryza sativa*) and maize (*Zea mays*) seeds, and serves as an energy reserve for various metabolic processes. Starch typically consists of two structurally-distinct components, viz. the basically linear α-1, 4-polyglucan amylose and highly branched α-1, 6-polyglucan amylopectin. Starch biosynthesis in the endosperm of cereals such as rice requires a concerted series of enzymatic reactions involving ADP-glucose pyrophosphorylase (AGPase), soluble starch synthase (SS), granule-bound starch synthase (GBSS), starch branching enzyme (BE), starch debranching enzyme (DBE), phosphorylase (PHO) and disproportionating enzyme (DPE). Several of these enzymes possess multiple isoforms which can be spatial- and temporal-specific (Ball et al. 1996; Smith et al. 1997; Ohdan et al. 2005; Hannah and James 2008; Hanashiro et al. 2008; Jeon et al. 2010). Other metabolic pathways involved in carbon flux can also modulate starch biosynthesis by providing various intermediate products such as hexose-phosphate and triose-phosphate.

In rice, mutants defective in these enzymes exhibit abnormal features of endosperm starch with opaque-kernel phenotypes, which were variously described as floury, glutinous, shrunken, dull, white-belly and white-core grains somewhere (Nelson and Pan 1995). As a result, these mutants provide valuable genetic materials for elucidation of metabolic processes related to nutrient storage during grain filling (Nelson and Pan 1995). For example, knockout of *OsSSIIIa* resulted in a decrease in long chain molecules, and a mutant endosperm was characterized by a loosely packed central portion exhibiting a floury-like phenotype (Ryoo et al. 2007). T-DNA insertion of the C4-type pyruvate orthophosphate dikinase gene (*OsPPDKB*) in a *floury endosperm*-*4* (*flo4*) rice mutant produced no corresponding transcript or protein, and white-core kernels in the mutant weighed about 6 % less than wild-type due to defective starch synthesis (Kang et al. 2005). In addition to biosynthesis enzymes, other factors might be indirectly related to starch synthesis. *FLO2*, which harbors a tetratricopeptide repeat motif, plays a pivotal regulatory role in both grain size and starch quality in rice by affecting storage starch accumulation in endosperm (She et al. 2010). *FLO6,* encoding a CBM48 domain-containing protein, is involved in compound granule formation and starch synthesis via direct interaction with isoamylase 1 (ISA1) in developing rice seeds (Peng et al. 2014).

Glycolysis is the predominant pathway supporting respiration in plants by generating various intermediate products, such as reductant and pyruvate (Plaxton 1996). Pyrophosphate: fructose-6-phosphate 1-phosphotransferase (PFP) catalyzes reversible interconversion between fructose-6-phosphate and fructose-1,6-bisphosphate, a rate-limiting step in the regulation of the primary carbohydrate metabolic flux toward glycolysis or gluconeogenesis (Basson et al. 2011). PFP utilizes pyrophosphate (PPi) as an alternative phosphoryl donor in place of ATP during the phosphorylation of Fru-6-P to Fru-1,6-P2 and this consequently provides an energy advantage to plants (Lim et al. 2009).

PFP typically works as a hetero-oligomer, comprising catalytic β- and regulatory α-subunits (Todd et al. 1995; Carlisle et al. 1990). In contrast to monocot species which contain only a single copy of *PFP*_*β*_ gene, dicot species generally have multiple isoforms (Wong et al. 1990; Botha and Botha 1991). Although several functions have been proposed for PFP, including roles in glycolysis, gluconeogenesis, equilibration of hexose-phosphate and triose-phosphate pools, modulation of PPi concentration during sucrose synthesis and degradation, and general adaptability to stresses (Theodorou et al. 1992; Hajirezaei et al. 1994; Mutuku and Nose 2012; Mustroph et al. 2013; Lim et al. 2013), its precise roles and significance in rice seed development are still obscure.

In the present study, we identified two allelic *PFP*_*β*_ rice mutants, designated as *pfp1*-*1* and *pfp1*-*2*, which were characterized by floury endosperm. Kernel thickness and starch content in the mutants were dramatically decreased, indicating that *PFP* plays a role in modulating carbon metabolism during grain filling.

## Materials and methods

### Plant materials and growth conditions

All rice seeds were from a long-term germplasm bank held at Nanjing Agricultural University (NAU). For map-based cloning, an F_2_ population was generated from crosses between the mutants and cv. N22 (*O. sativa* L. ssp. *indica*) and individuals showing the floury phenotype were used for DNA extraction and genotyping. *Japonica* cv. Kitaake and the *pfp1*-*1* mutant were used for genetic transformation acceptors via the *Agrobacterium tumefaciens*-mediated transformation method (Hiei et al. 1994). Plants were grown in a paddy field under natural conditions at NAU.

### Scanning electron microscopy (SEM)

Samples were prepared as previously described (Kang et al. 2005). Briefly, ten mature rice seeds were husked, transversely cut by a knife and coated with gold by E-100 ion sputter. The mounted specimens were then observed using a HITACHI S-570 scanning electron microscope at an acceleration voltage of 20 kV.

### Measurement of starch content

Rice grains were husked and ground to flour consistency in a mill. Starch contents of 100 mg flour were enzymatically measured with a total starch assay kit (Megazyme, Ireland) following the manufacturer’s recommendations.

### Metabolite determination

Levels of seed soluble monosaccharides (sucrose, glucose and fructose) were measured by gas chromatography mass spectrometry (GC–MS) analysis as previously reported (Tan et al. 2010; Li et al. 2013) with some modifications. Briefly, 200 mg flour samples were dissolved in 2 mL Me_2_SO, mixed with 150 μL of acetic anhydride and 30 μL of 1-methylimidazole, and stirred for 10 min in glass tubes; 600 μL of double-distilled H_2_O (ddH_2_O) was added to each tube to remove excess acetic anhydride, and 100 μL of CH_2_Cl_2_ was added to extract the acetylated derivatives. The tubes were centrifuged for 1 min to separate organic phase. Finally, 1 μL of the lower methylene chloride layer was injected for GC–MS analysis.

### Map-based cloning of the mutant gene

Genomic DNA was extracted according to the CTAB method (1.5 % CTAB, 75 mM Tris–HCl pH 8.0, 15 mM EDTA pH 8.0, 1.05 M NaCl). For genetic linkage analysis, 10 F_2_ progeny exhibiting floury endosperm phenotype from a cross of *pfp1* and N22 were genotyped with 176 genome-wide simple sequence repeat (SSR) markers showing polymorphism between the two parents. For fine mapping, de novo SSR and cleaved amplified polymorphic sequence (CAPS) molecular markers were developed from comparisons of genome sequences annotated by the National Center for Biotechnology Information (NCBI). Details of the new markers are provided in Supplemental Table 1. The PCR procedure was carried out as follows: 95 °C for 5 min, followed by 32 cycles of 94 °C for 30 s, annealing for 30 s, 72 °C for 40 s, and a final elongation step at 72 °C for 5 min. cDNAs of the ten *open reading frames* (*ORFs*) in the fine-mapped region were amplified from wild-type and mutants seeds, and sequenced.

### Genetic complementation assay

cDNA of candidate gene was amplified by primer pair PFP_β_-OE (Supplemental Table 1) and cloned into the binary vector pCUbi1390 driven by the ubiquitin promoter. The derived PFP_β_-OE construct was then introduced into *calli* generated from the *pfp1*-*1* mutant. To confirm positive transgenic plants, leaves of *pfp1*-*1* mutant and T_0_ transgenic plants were incubated in water containing hygromycin of 50 mg/L for 1 week. 23 individuals that showed resistance to hygromycin were considered as possible positive transformants. Furthermore, specific primers PFP_β_-OE –IN (Supplemental Table 1) derived from plasmid constructs was used to confirm the results. The PCR procedure was carried out as follows: 95 °C for 5 min, followed by 34 cycles of 94 °C for 30 s, 55 °C for 30 s, 72 °C for 1 min, and a final elongation step at 72 °C for 5 min.

### RNA extraction and qPCR analysis

Total RNA were extracted from various tissues of wild-type (WT) and the two mutants using an RNAprep pure Plant Kit (TIANGEN, Beijing), and treated with DNase I following the manufacturer’s recommendations. First-strand cDNA was synthesized with oligo (dT_18_) based on a PrimeScript Reverse Transcriptase Kit (Takara, Japan). Real-time PCR was performed on an ABI7500 real-time PCR system using SYBR Premix Ex Taq (Takara) with rice *Ubiquitin* as an endogenous control. Relative expression levels of specific genes were quantitated from three biological replicates via the $2^{{ - {\Delta \Delta }C_{\text{t}} }}$ method (Livak and Schmittgen 2001). All primer pairs for expression analysis are listed in Supplemental Table 1.

### Phylogenetic analysis

Amino acid sequences of PFP homologous proteins were obtained from National Center for Biotechnology Information (NCBI), and the phylogenetic tree was constructed based on the neighbor-joining method using MEGA 5.0 software. Bootstrap values were estimated from bootstrap analysis of 1000 replicates.

### Subcellular localization

The coding sequence of PFP_β_ was amplified by primer pair PFP_β_-GFP (Supplemental Table 1) and fused to the N-terminus of GFP under control of the CaMV35S promoter in the transient expression vector pAN580. This construct, referred to as PFP_β_-GFP, was introduced into rice protoplasts according to protocols described previously (Zhang et al. 2011). After incubation in the dark at 28 °C for 16 h, the GFP signal was observed using a confocal laser scanning microscope (LSM 700, Carl Zeiss).

### Histochemical beta-glucuronidase (GUS) staining

A putative 2 kb promoter fragment upstream of the ATG start codon was cloned with primer pair PFP_β_-GUS (Supplemental Table 1) and fused into the binary vector pCAMBIA1305 to generate the plasmid p*proPFP*:GUS for genetic transformation of *calli* of Kitaake. Various tissues of 5 T_1_ positive lines were incubated in staining buffer (1 mg mL^−1^ X-Gluc, 50 mM sodium phosphate buffer, pH 7.0, 1 mM potassium ferrocyanide, 1 mM potassium ferricyanide, 0.1 % Triton X-100, 10 mM EDTA, pH 8.0) at 37 °C in the dark followed with incubation in 100 % EtOH to remove chlorophyll.

### Enzyme activity measurement

Rice seeds of DJY and the two mutants were homogenized in liquid nitrogen and 200 mg of the grounded tissues were used for protein extraction and PFPase activity measurement according to a previous study (Van der Merwe et al. 2010). The enzyme activity assay was performed at 25 °C, and one unit of PFP activity was defined as the formation of 1 μmol of NAD^+^ per minute.

### Three-dimensional structure prediction for PFP

The PFP sequence was submitted to the Swiss-Model homology modeling server (http://swissmodel.expasy.org/SWISS-MODEL.html) to predict its three-dimensional structure via the automatic modeling mode (Arnold et al. 2006).

## Results

### Characterization of *pfp* mutants

**Fig. 1:**
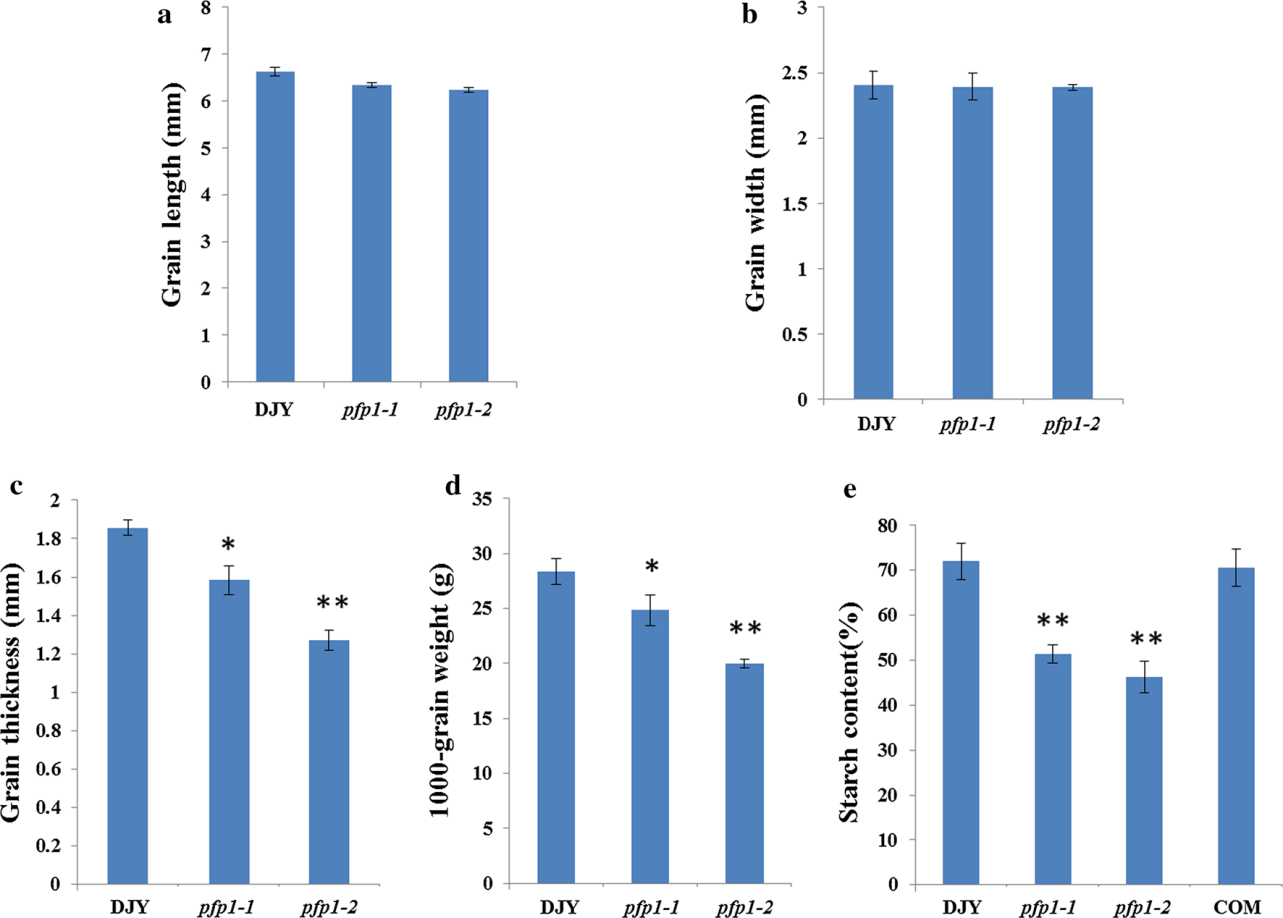
Grain phenotypes of wild-type (DJY), *pfp1*-*1*, *pfp1*-*2* and a representative complementation line (COM). **a** Grain length; **b** Grain width; **c** Grain thickness; **d** 1000-grain weight; **e** Starch content. Values are presented as mean ± SD; Statistically significant differences compared with DJY samples were determined by Student’s *t* test (*P < 0.05; **P < 0.01). At least 20 grains were analyzed for the measurement of grain length, width and thickness and 3 biological replications were used in the determination of 1000-grain weight and starch content

Both mutants (*pfp1*-*1*and *pfp1*-*2*) exhibiting floury endosperm phenotypes were identified from an ethyl methane sulfonate (EMS) chemical mutant pool of *japonica* rice cv. DJY. The mutants showed no visible differences from wild-type (WT) during the vegetative and reproductive stages, with normal plant height, tiller number and panicle architecture (Supplemental Fig. 1). The grain length and width of the mutants were similar to WT but grain thickness was dramatically decreased, and the grain of *pfp1*-*2* was only 68 % of WT in thickness (Fig. 1a–c). Accordingly, the 1000-grain weights of *pfp1*-*1*and *pfp1*-*2* were greatly reduced by 4.9 and 9.6 g, respectively (Fig. 1d). Additionally, starch contents of *pfp1*-*1* and *pfp1*-*2* were also significantly reduced by 20–26 %, respectively, compared with WT (Fig. 1e).

### The endosperms of *pfp1*-1and *pfp1*-2 were abnormally packed

**Fig. 2:**
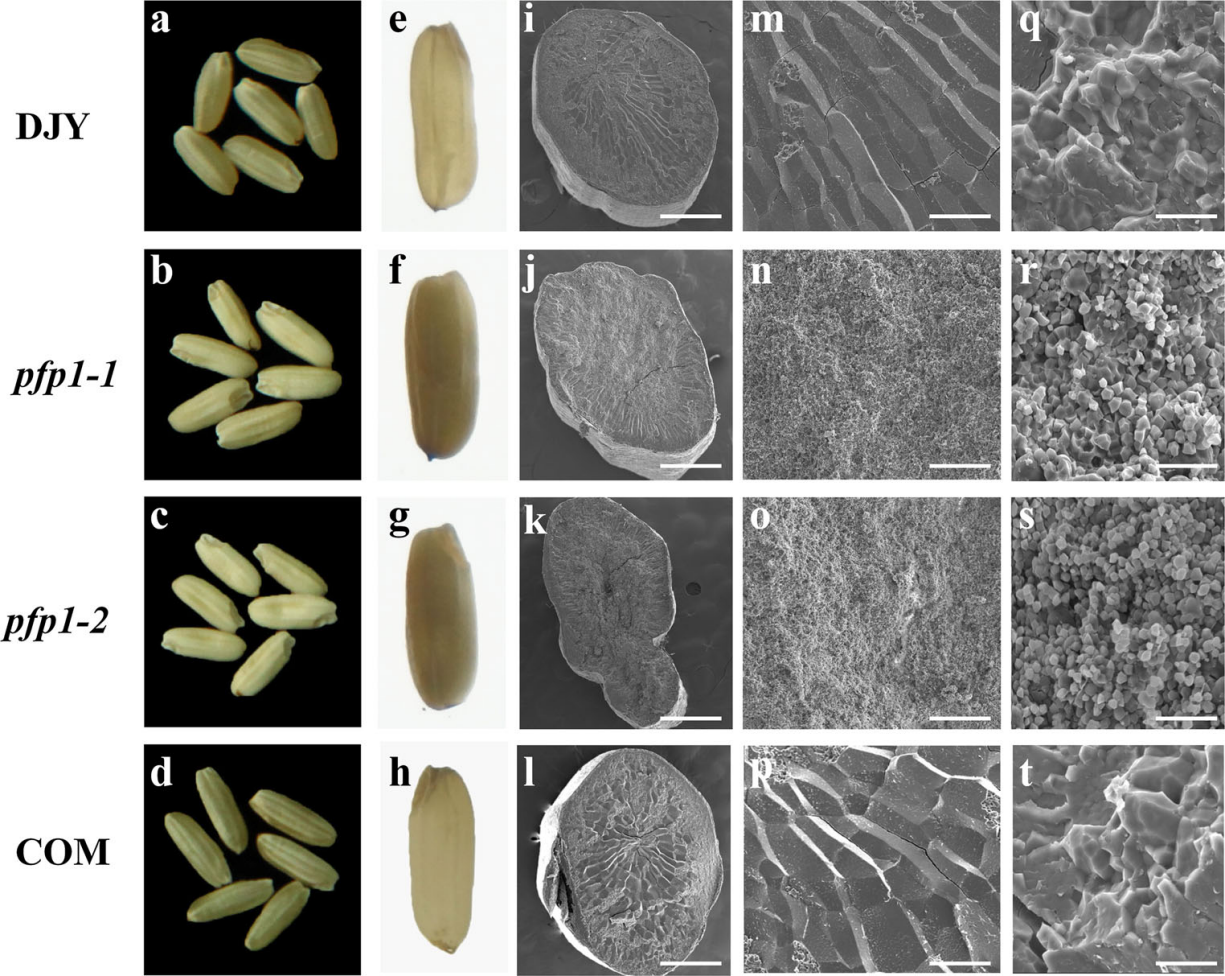
Seed morphologies and scanning electron microscopy (SEM) of transverse sections of endosperm. **a**–**h** Seed morphologies of DJY (WT), *pfp1*-*1*, *pfp1*-*2* and COM (representative complementation line) under natural light (**a**–**d**) and bright-field illumination (**e**–**h**); **i**–**t** SEM of transverse sections of the endosperms of DJY, *pfp1*-*1*, *pfp1*-*2* and COM at enlargements of **i**–**l** ×4, (*bars* 0.75 mm); **m**–**p** ×200 (*bars* 15 μm); **q**–**t** ×2000 (*bars* 150 μm)

Husked grains of both *pfp1*-*1* (Fig. 2b, f) and *pfp1*-*2* (Fig. 2c, g) were opaque under bright-field illumination, in contrast to the translucent phenotype of WT (Fig. 2a, e). SEM of transverse sections revealed that, unlike the regular, compact crystal structure of WT (Fig. 2l, m, q), the endosperms of both *pfp1*-*1* (Fig. 2j, n, r) and *pfp1*-*2* (Fig. 2k, o, s) consisted of small, spherical and loosely packed starch granules with large air spaces, in agreement with their floury-like endosperms.

### Map-based cloning of the mutant alleles

**Fig. 3:**
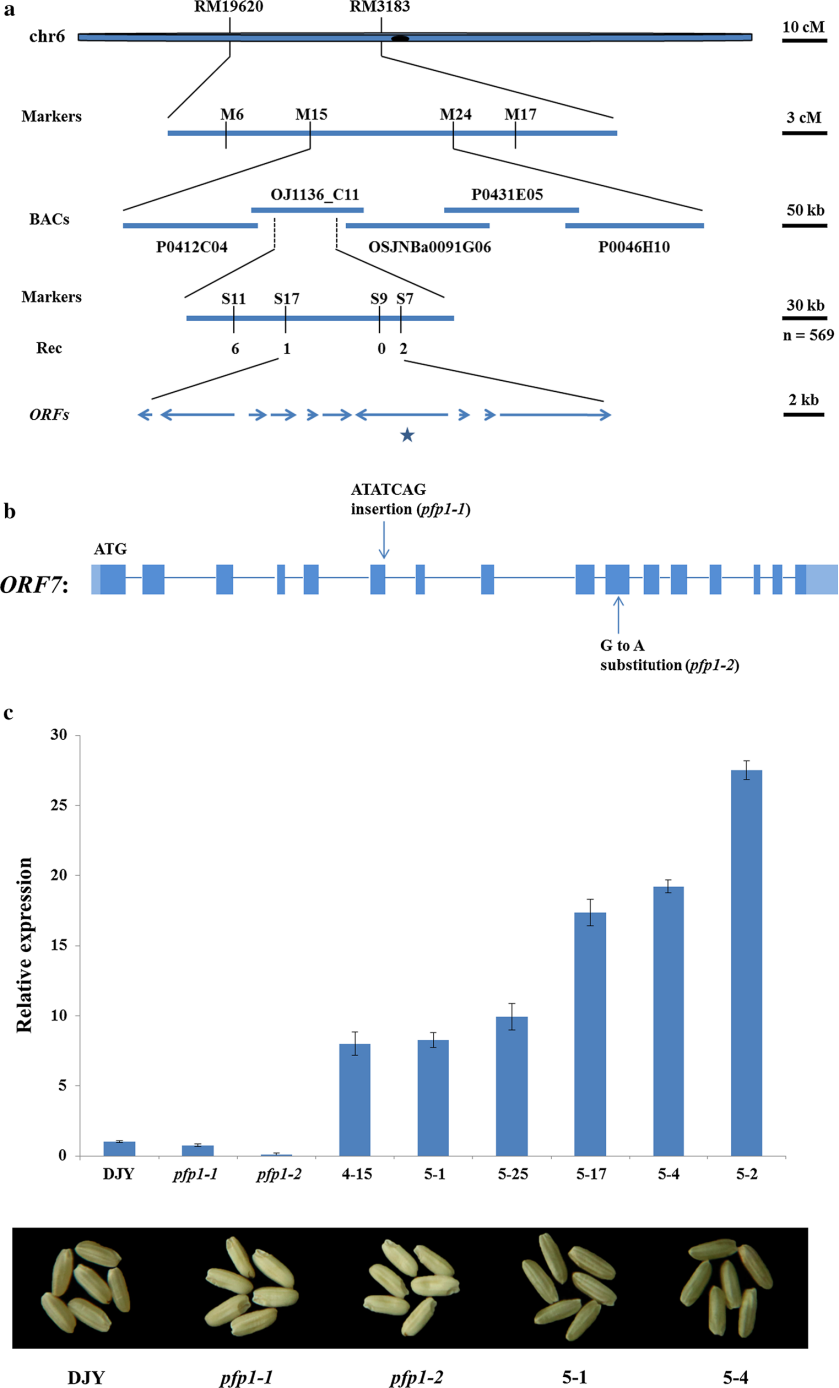
Map-based cloning and genetic complementation of the *pfp1* mutant gene. **a** The *pfp1*-*1* mutant locus was initially mapped to the short arm of chromosome 6 between molecular markers RM19620 and RM3183, and further narrowed to a 68 kb region on BAC OJ1136_C11, containing ten open reading frames (*ORFs*). The markers and numbers of recombinants (Rec) are indicated. *Asterisk* denotes the candidate gene; **b** Schematic representation of *Pyrophosphate: fructose*-*6*-*phosphate 1*-*phosphotransferase* (*PFP*). A G to A substitution was presented in the tenth exon of *pfp1*-*1* and a 7 bp (ATATCAG) insertion is present in the splicing site of the sixth exon of *pfp1*-*2*. Exons encoding the protein are *dark blue*, 5′ and 3′ UTR regions are *light blue*; **c** the floury endosperm phenotype was rescued by introduction of wild-type cDNA. *Upper panel* indicated relative expression of *PFP1* in grains of different transgenic plants, and *lower panel* shows representative seeds. Values are presented as mean ± SD of four replications

To identify the mutant locus, map-based cloning was performed. Ten progeny exhibiting floury endosperm were selected from an F_2_ population (*pfp1* × N22) and genotyped with 176 genome-wide SSR and Indel markers which exhibited polymorphism between the two parent lines. The mutant phenotype was associated with SSR markers RM586 and RM225 on the short arm of chromosome 6. Subsequent fine mapping based on 569 progeny narrowed the mutant locus to a 68 kb region with 10 *ORFs* (Fig. 3a). DNA sequencing identified that a G to A substitution in the 10th exon of *ORF7* (*LOC_Os06g13810*) happened, resulting in a D_394_ to N_394_ transition in *pfp1*-*1* (Fig. 3b). In *pfp1*-*2*, a 7 base (ATATCAG) insertion was present in the splicing site of the 6th exon, leading to a premature stop codon (Fig. 3b).

To confirm that *ORF7* was responsible for the floury phenotype, we infused the 1.7 kb of wild-type cDNA into the binary vector pCUbi1390 under control of the maize ubiquitin promoter and introduced the resulting recombined vector into mutant *calli* via *Agrobacterium* -mediated transformation. 23 positive transformants were selected with recombined vector-specific primers, and expression levels were determined by quantitative PCR (qPCR). Grains from six positive transgenic plants showed various elevated transcription levels (ranging from 8- to 18-fold higher than the control) and had fully rescued non-floury endosperm phenotypes (Figs. 2d, h, 3c). The starch contents (Fig. 1e) and crystal structures of the starch granules (Fig. 2l, p, t) were restored to wild-type levels, indicating that the mutations in *ORF7* were indeed the cause of the mutant phenotypes.

### *ORF7* encoded the PFP β subunit

**Fig. 4:**
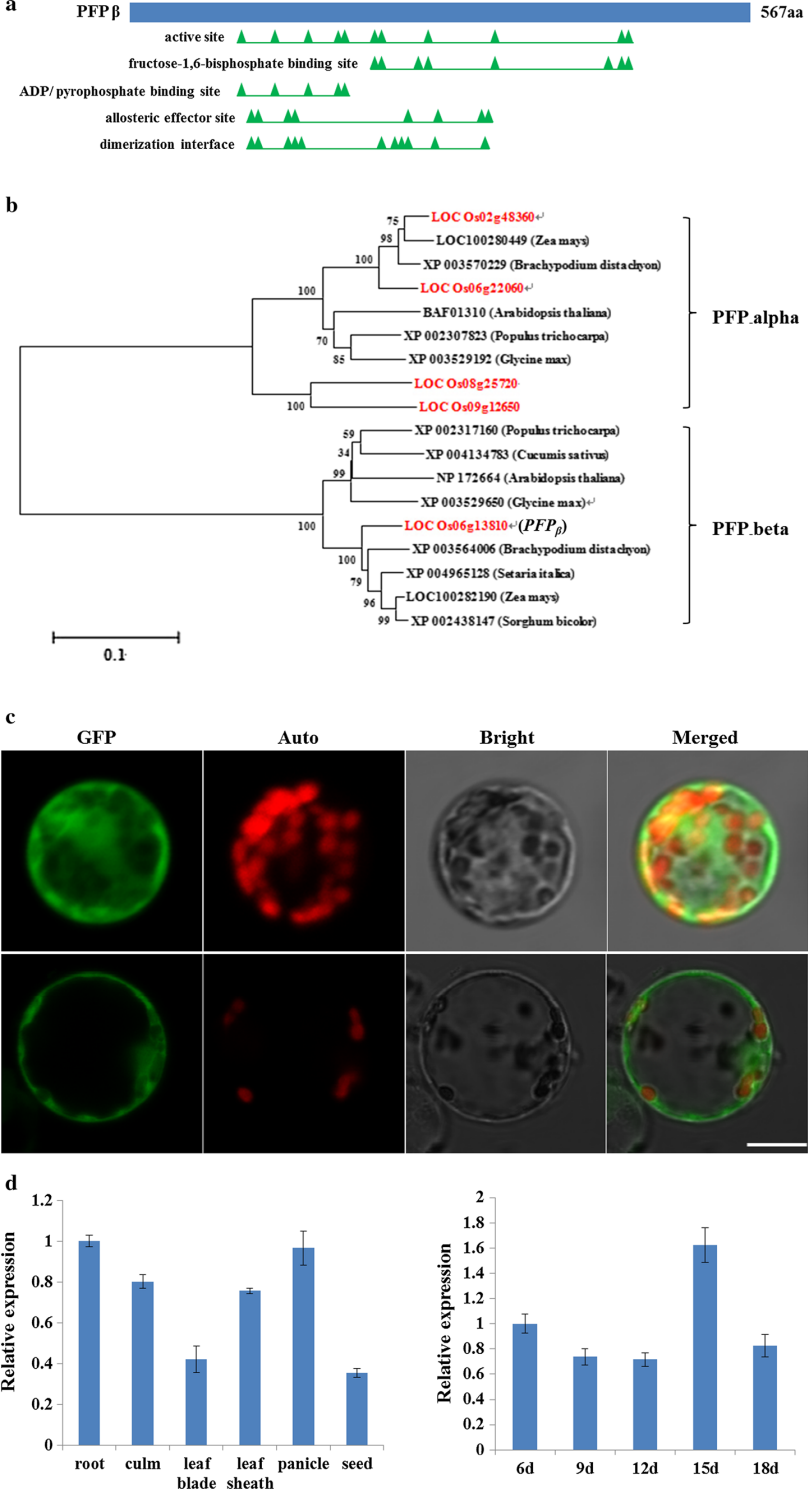
Functional domain prediction, phylogenetic analysis, subcellular localization and expression pattern of *PFP*_*β*_. **a** Deduced functional sites in PFP_β_ (*green triangles*); **b** phylogenetic analysis of PFP_α_ and PFP_β_. The topology of this tree was generated by the neighbor-joining method with MEGA 5.0 software. *Scale bar* shows the number of nucleotide substitutions per site; **c** subcellular localization of PFP_β_. *Upper panel* shows GFP fluorescence and *lower panel* shows PFP_β_-GFP distribution. GFP, *auto* and *bright* indicate GFP fluorescence of GFP or PFP_β_-GFP, chloroplast autofluorescence and bright-field respectively. *Bar* indicates 10 μm; **d** Expression pattern of *PFP*_*β*_ detected by qPCR with *ubiquitin* as the endogenous control for data normalization. *Left* *PFP*_*β*_ expression analysis in various tissues; *right* *PFP*_*β*_ expression in seeds at different developmental stages in days post anthesis. Values are mean ± SD (*n* = 4)

*ORF7* encodes a PFP β subunit with 567 amino acid residues; it contains an active site, fructose-1,6-bisphosphate binding site, ADP/pyrophosphate binding site, allosteric effector site and a dimerization interface (Fig. 4a). PFP belongs to the phosphofructokinase (PFK) superfamily, which catalyzes critical steps in the glycolytic pathway (Supplemental Fig. 2) and is extensively distributed among the plant kingdom (Fig. 4b). Phylogenetic analysis showed four copies encoding the regulatory α-subunits were present in the rice genome, however, only one copy of *ORF7* (*LOC_Os06g13810*) encoding the catalytic β-subunit existed (Fig. 4b), indicating the essential role of ORF7 (PFP_β_) for PFP activity.

### Subcellular localization and expression pattern of *PFP*_*β*_

To determine the subcellular localization of PFP_β_ protein, a transient expression assay was performed with the rice protoplasts. Full length of *PFP*_*β*_ was fused to the N-terminus of GFP, and confocal microscopy observation of PFP_β_-GFP localization in isolated protoplasts showed that PFP_β_ was localized in cytoplasm, just as the case of free GFP, which showed the dispersed distribution throughout the protoplast (Fig. 4c).

In wild-type plants, qPCR demonstrated that *PFP*_*β*_ was ubiquitously expressed in various tissues, including roots, culms, leaf blades, leaf sheaths, panicles and developing seed (Fig. 4d). Further examination revealed that the transcripts of *PFP*_*β*_ could be detected in both early (6 days after flowering) and late phases (18 days after flowering) of starch accumulation during grain filling (Fig. 4d). Spatial expression pattern of *PFP*_*β*_ was further examined in transgenic plants expressing the *GUS* reporter gene under the control of the *PFP*_*β*_ promoter. Histochemical analysis of T_1_ positive p*proPFP*_*β*_:GUS transgenic plants showed that the *PFP*_*β*_ promoter was active in various tissues (Supplemental Fig. 2), which was consistent with our qPCR analysis.

### Decreased PFP activity influenced carbon metabolism

**Fig. 5:**
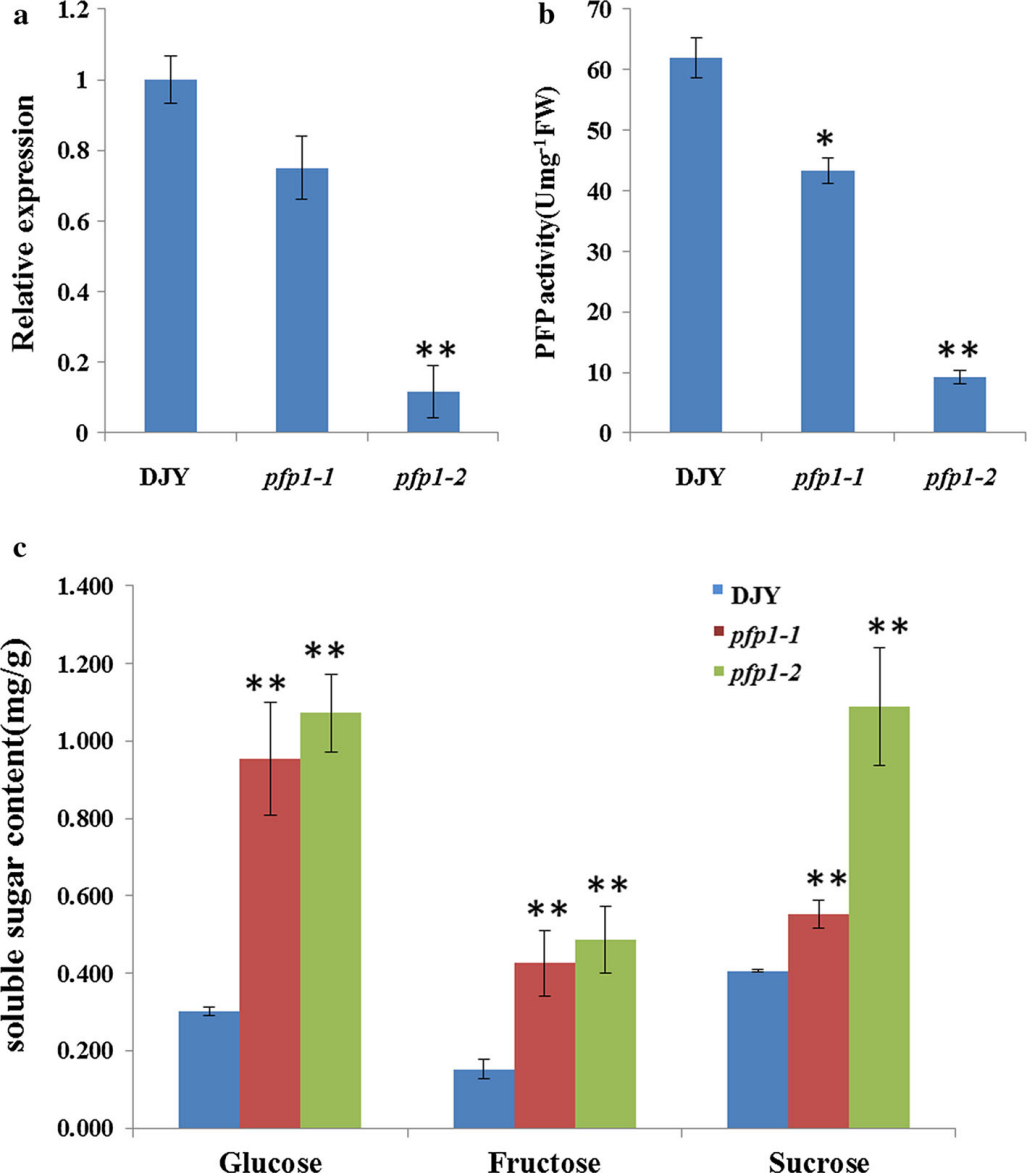
*PFP*_*β*_ expression analysis, enzyme activity assay and soluble sugar content. **a** Expression levels of *PFP*_*β*_ detected by qRT-PCR; **b** PFP activity assays; **c** glucose, fructose and sucrose contents of developing seeds at 12 days post anthesis. Values are presented as mean ± SD of three biological replications; statistically significant differences compared to DJY samples were determined by Student’s *t* test (*P < 0.05; **P < 0.01)

Compared with wild-type plants, *PFP*_*β*_ expression level in the grain was slightly decreased in the *pfp1*-*1* mutant, but highly reduced in *pfp1*-*2* due to the 7 bp insertion at the splicing site (Fig. 5a). Consistent with this, PFP enzyme activities in *pfp1*-*1* and *pfp1*-*2* in the glycolysis direction reached 69.8 and 15 % of that in wild-type, respectively (Fig. 5b). Soluble sucrose, glucose, and fructose levels were also significantly higher in both *pfp1*-*1* and *pfp1*-*2* (Fig. 5c). Meanwhile, the expression of 35 genes associated with starch biosynthesis was analyzed to investigate their possible regulations by *PFP*. The results showed that, except for *OsAGPS2a*, *OsPUL* and *α*-*amylase 3E,* most of the tested genes in *pfp2* were significantly upregulated as compared to wild-type (Supplemental Fig. 3). We concluded that PFP_β_ modulated the equilibration of cellular carbon metabolism for starch biosynthesis, thereby affecting the grain filling process.

## Discussion

Starch, as the primary storage substance, occupies most compartments of the rice seed endosperm (Hannah and James 2008; Jeon et al. 2010). Defects in rice starch forming affect seed filling and further reduce both yield and quality. In the present study, we identified two allelic mutants exhibiting floury endosperm phenotypes caused by loosely packed starch granules. Map-based cloning revealed that the *PFP*_*β*_ gene encodes the β subunit of PFP, which catalyzes a rate-limiting step in glycolysis (Mutuku and nose 2012; Mustroph et al. 2013).

Both mutants grew normally, but exhibited abnormal floury endosperm. Compared to *pfp1*-*1*, *pfp1*-*2* showed a more severe defective phenotype with significantly decreased grain thickness and grain weight (Figs. 1c, d, 2c, k). According to modeling analysis, we speculated that the D_394_ to N_394_ transition in *pfp1*-*1* produced a minor alteration in molecular configuration, causing only a modest reduction in enzyme activity (Supplemental Fig. 4). However, the premature stop codon in *pfp1*-*2* represented a functional mutation that almost abolished its enzyme activity (Supplemental Fig. 3; Fig. 5a, b). In parallel with this, the accumulation of soluble sugars (sucrose, fructose and glucose) was far higher in *pfp1*-*2* than those in *pfp1*-*1* (Figs. 1e, 5c).

Abnormal starch accumulation in early seed development stages, but with progress to normal ripening in later stages, generally results in white-core endosperm phenotypes, or white-belly mutant morphologies (Nelson and Pan 1995; Kang et al. 2005; Ryoo et al. 2007). Expression pattern analysis showed that *PFP*_*β*_ was continuously expressed during grain filling (Fig. 4d) and, meanwhile, dysfunction of PFP_β_ resulted in a floury endosperm phenotype (Fig. 2), implying that *PFP*_*β*_ functions in both the early and late phases of starch accumulation during grain filling in rice.

In rice, mutants defective in starch biosynthesis-related enzymes mostly exhibit abnormal features in stored starch, yet other factors might also work indirectly. For example, failure to express a protein disulphide isomerase-like protein caused endoplasmic reticulum stress and affected the activities of several starch synthesis-related enzymes such as soluble starch synthase I and ADP-glucose pyrophosphorylase, leading to a floury endosperm phenotype (Han et al. 2012). Elevated expression of *chalk5,* encoding a vacuolar H^+^-translocating pyrophosphatase (V-PPase), disturbed the pH homeostasis of the endomembrane trafficking system in developing seeds and resulted in grain chalkiness (Li et al. 2014). In our mutant plants, grains are shrunken in thickness, and starch contents were dramatically reduced by ~25 % (Fig. 1e), indicating that biosynthesis of storage starch is greatly compromised by *PFP*_*β*_ mutation. Carbon is originally fixed by photosynthesis process and transported into grains in the form of soluble sucrose. After entering the cells, sucrose degrades into fructose and glucose, and the latter undergoes the glycolysis pathway for further degradation to provide hexose-P, triose-P and various intermediate products (Plaxton 1996). Intriguingly, we found that the levels of soluble sugars like sucrose, fructose and sucrose were all significantly increased (Fig. 5c), suggesting inefficient carbon flux in the direction of starch biosynthesis. Furthermore, qPCR analysis showed that the expression patterns of starch synthesis-related genes differed depending on the individual genes (Supplemental Fig. 3). Overall, most of the genes are down-regulated, such as AGPS2a, BEI, ISA2, PUL, Flo2, Amylase 3E and PGI-b, suggesting that *PFP*_*β*_ may regulate starch biosynthesis by affecting the activities of these enzymes. Similar results were reported for the *flo2*, *chalk5* and *flo6* mutants (She et al. 2010; Li et al. 2014; Peng et al. 2014). Taken together, we conclude that PFP_β_ regulates starch biosynthesis in rice grains by affecting carbon flux and activities of starch synthesis-related enzymes. In transgenic tobacco plants severe decreases in the expression level and activity of PFP resulted in no visible lesions on plant growth or major changes in carbon fluxes, implying that significantly reduced PFP activity could be compensated by changes in Fru2, 6-bisP (Nielsen and Stitt 2001). Transgenic *Arabidopsis* plants that overexpressed either of the PFP α or β subunits displayed increased PFP activity and slightly faster growth relative to wild-type plants. In contrast, RNAi lines showed significantly retarded growth consistent with the decreased PFP activity. However, no detectable change in the carbon partitioning profile in the leaves of the transgenic *Arabidopsis* plants was observed. The growth retardation of the RNAi lines was accompanied by reduced CO_2_ assimilation rates, suggesting that *PFP* might be also involved in other cellular processes in addition to carbon metabolism (Lim et al. 2009, 2013). In transgenic sugarcane, clones with 45–95 % reduced PFP activity displayed no visual phenotypic effects, but had significantly increased sucrose concentrations and hexose-phosphate : triose-phosphate ratios during internode development (Van der Merwe et al. 2010). In our study, though *PFP*_*β*_ was ubiquitously expressed in the various tissues tested (Fig. 4d; Supplemental Fig. 2), no significant phenotypic lesions during vegetative and reproductive stages, such as plant height, tiller number, heading date and panicle architecture (Supplemental Fig. 1), were observed in two *pfp* mutants. It is presumed that other factors compensate for the roles of *PFP*_*β*_ during vegetative growth in rice, as described in transgenic tobacco and sugarcane (Nielsen and Stitt 2001; Van der Merwe et al. 2010). However, the 1000-grain weight was dramatically reduced by 12.5 and 29.5 %, and the storage starch content was reduced by ~25 % (Fig. 1d, e), suggesting that *PFP*_*β*_ is indispensable for accumulation of starch in endosperm. In summary, our work here provides clues suggesting that *PFP*_*β*_ has a pivotal role in the accumulation of starch in the endosperm and sets a foundation for understanding the regulation of grain yield and quality in rice.

### **Author contribution statement**

E. C. Duan, Y. H. Wang, X. Zhang, X. P. Guo, L. Jiang and J. M. Wan designed the research; E. C. Duan, Y. H. Wang, J. P. Zhu, M. S. Zhong, H. Zhang and B. X. Ding performed the research; E. C. Duan, Y. H. Wang, L. L. Liu, L. Jiang and J. M. Wan wrote the paper.

## Electronic supplementary material

Below is the link to the electronic supplementary material.

Supplementary material 1 (DOCX 6404 kb)

## Abbreviations

EMS: Ethyl methanesulfonate
SEM: Scanning electron microscopy
GC–MS: Gas chromatography mass spectrometry
SSR: Simple sequence repeat
Indel: Insertion and deletion
cDNA: Complementary DNA
ORF: Open reading frame
qPCR: Quantitative polymerase chain reaction
