# Supplementary material for: Pyrophosphate: fructose-6-phosphate 1-phosphotransferase (PFP) regulates carbon metabolism during grain filling in rice
Source: Plant Cell Rep. 2016 Mar 18;35:1321–31. doi: 10.1007/s00299-016-1964-4 (PMC4869756; doi:10.1007/s00299-016-1964-4)
Supplement: Supplementary file 1 — Supplementary material 1 (DOCX 6404 kb) [file 299_2016_1964_MOESM1_ESM.docx]

###### Supplementary Information

###### Supplemental table 1, Supplemental figures 1-5

**Erchao Duan^1a^** **• Yihua Wang^1a^ • Linglong Liu^1^ • Jianping Zhu^1^ • Mingsheng Zhong^1^ • Huan Zhang^1^ • Sanfeng Li^2^ • Baoxu Ding^1^ • Xin Zhang^3^ • Xiuping Guo^3^ • Ling Jiang^1^ • Jianmin Wan^1, 3^**

**pyrophosphate: fructose-6-phosphate 1-phosphotransferase (PFP) regulates carbon metabolism during grain filling in rice**

**E.C. Duan^1^ • Y.H. Wang^1^• L.L. Liu^1^ • J.P. Zhu^1^ • M.S. Zhong^1^ • H. Zhang^1^ • S.F. Li^2^ • B.X. Ding^1^ • X. Zhang^3^ • X.P. Guo^3^ • L. Jiang^1^ • J.M. Wan^1, 3^**

*^1^National Key Laboratory for Crop Genetics and Germplasm Enhancement, Jiangsu Plant Gene Engineering Research Center, Nanjing Agricultural University, Nanjing 210095, China*

*^2^State Key Laboratory of Rice Biology, China National Rice Research Institute, Chinese Academy of Agricultural Sciences, Hangzhou 310006, China*

*^3^National Key Facility for Crop Gene Resources and Genetic Improvement, Institute of Crop Science, Chinese Academy of Agricultural Sciences, Beijing 100081, China*

* Corresponding authors:

Jianmin Wan

Telephone: +86-25-84396516

Fax: +86-25-84396516

E-mail: wanjm@njau.edu.cn

^a^These authors contributed equally to this work.

**Supplemental table 1.** Sequences of primers used in this study

| **Primer name** | **Forward sequence** | **Reverse sequence** | **Use** |
| --- | --- | --- | --- |
| M6 | GCGATGTCTTCGAGTCCC | CCTATGAACAGTGTATCAACCC | Mapping |
| M15 | TTGCTTACCAGCGCATTCA | CCCAGGCTTCACCAGACAT | Mapping |
| M17 | AATCGTACTTCTGGACGACGGG | CGCCTGGTTGGCTTTTGC | Mapping |
| M24 | GCGATGTGCATGGTACTC | TGGCAGGTCACATAAGCT | Mapping |
| S7 | TCTTTGTTTACGGTACTTCA | CGAAGGGTGACTTTTACA | Mapping |
| S9 | CGACGACGCAGGTGAGGA | CTCGGTTCAATTCATTCATCCA | Mapping |
| S11 | ATAGATAGATAGATAATAGAT | GCAATCGTTGCCAAGATA | Mapping |
| S17 | TTGCGGAAGGTGGTAGAG | CCTCGGTTCAATTCATTCA | Mapping |
| PFP | GTTGGTGGAACTGCATTGAC | AGCCTTCTTGATCACTGGCT | qPCR |
| OsAGPS1 | GTGCCACTTAAAGGCACCATT | CCCACATTTCAGACACGGTTT | qPCR |
| OsAGPS2a | ACTCCAAGAGCTCGCAGACC | GCCTGTAGTTGGCACCCAGA | qPCR |
| OsAGPS2b | AACAATCGAAGCGCGAGAAA | GCCTGTAGTTGGCACCCAGA | qPCR |
| OsAGPL1 | GGAAGACGGATGATCGAGAAAG | CACATGAGATGCACCAACGA | qPCR |
| OsAGPL2 | AGTTCGATTCAAGACGGATAGC | CGACTTCCACAGGCAGCTTATT | qPCR |
| OsAGPL3 | AAGCCAGCCATGACCATTTG | CACACGGTAGATTCACGAGACAA | qPCR |
| OsAGPL4 | TCAACGTCGATGCAGCAAAT | ATCCCTCAGTTCCTAGCCTCATT | qPCR |
| OsSSI | GGGCCTTCATGGATCAACC | CCGCTTCAAGCATCCTCATC | qPCR |
| OsSSIIa | GCTTCCGGTTTGTGTGTTCA | CTTAATACTCCCTCAACTCCACCAT | qPCR |
| OsSSIIb | TAGGAGCAACGGTGGAAGTGA | GTGAACGTGAGTACGTGACCAAT | qPCR |
| OsSSIIc | GACCGAAATGCCTTTTTCTCG | GGGCTTGGAGCCTCTCCTTA | qPCR |
| OsSSIIIb | ATTCCGCTCGCAAGAACTGA | CAACCGCAGGATAACGGAAA | qPCR |
| OsSSIVa | GGGAGCGGCTCAAACATAAA | CCGTGCACTGACTGCAAAAT | qPCR |
| OsSSIVb | ATGCAGGAAGCCGAGATGTT | ACGACAATGGGTGCCAAGAT | qPCR |
| OsGBSSI | AACGTGGCTGCTCCTTGAA | TTGGCAATAAGCCACACACA | qPCR |
| OsGBSSII | AGGCATCGAGGGTGAGGAG | CCATCTGGCCCACATCTCTA | qPCR |
| OsBEI | TGGCCATGGAAGAGTTGGC | CAGAAGCAACTGCTCCACC | qPCR |
| OsBEIIa | GCCAATGCCAGGAAGATGA | GCGCAACATAGGATGGGTTT | qPCR |
| OsBEIIb | ATGCTAGAGTTTGACCGC | AGTGTGATGGATCCTGCC | qPCR |
| OsISA1 | TGCTCAGCTACTCCTCCATCATC | AGGACCGCACAACTTCAACATA | qPCR |
| OsISA2 | TAGAGGTCCTCTTGGAGG | AATCAGCTTCTGAGTCACCG | qPCR |
| OsISA3 | ACAGCTTGAGACACTGGGTTGAG | GCATCAAGAGGACAACCATCTG | qPCR |
| OsPUL | ACCTTTCTTCCATGCTGG | CAAAGGTCTGAAAGATGGG | qPCR |
| OsPHOL | TTGGCAGGAAGGTTTCGCT | CGAAGCCTGAAGTGAACTTGCT | qPCR |
| OsPHOH | CACCAAGACGAAGCTCATCAAG | TTCACTCGTTGCTGGGTTCTC | qPCR |
| OsDPE2 | CAAGTACACCACAAGACCAGCAA | CGTCCAACAGCGAATCCAAT | qPCR |
| Flo2 | CACACCCTCCAGCAATATCA | CCTTCTGCGACTGCTTTTCT | qPCR |
| Flo4 | CATGCACTGTTCGAGGAGAA | GGGAAATGGCTCTCCCTTAG | qPCR |
| SS1 | AATGGTATCCTCCGCAAGTG | GGCTTGCATTTCCCTCATAA | qPCR |
| SS2 | GCTGAAGGACAGGAACAAGC | CACCACAGACAACCACAAGG | qPCR |
| SS3 | CATGTACCCCCTGCTCAACT | GTCAGCTGTAATGCCTGCAA | qPCR |
| α-amylase 3C | CTGGCTCCACACAGAACTCA | CGTAGACATCTCCGTCAGCA | qPCR |
| α-amylase 3E | ACAAGGTCATGCAGGGCTAC | GTTCCTTGACCGGATTTCAG | qPCR |
| PGIa | GTTGCACGAAGCATCAAAGA | ATGTTTCGCAACAGCATCAG | qPCR |
| PGIb | GCCAAAGTCAGAGCCAAAAG | GAACCAATGTGCAACACCAG | qPCR |
| Ubiquitin | GCTCCGTGGCGGTATCAT | CGGCAGTTGACAGCCCTAG | qPCR |
| PFP_β_-GUS | CCATGATTACGAATTCGGCGG  GCGCCGAGGCCGTGG | CTCAGATCTACCATGGGCGAAG  CGAGATTTGGTTTG | GUS  staining |
| PFP_β_-GFP | CGGTCCCGGGGGATCCATGG CGGCGGCGGCGGTGGC | TGCTCACCATGGATCCTGCCTC GGCGCCGAGTTCCA | Subcellular localization |
| PFP_β_-OE  PFP_β_-OE-IN | TTACTTCTGCACTAGGTACCA TGGCGGCGGCGGCGGTGGC  GGGAAGACAGGACTGATTAC | GAATTCCCGGGGATCCCTAT GCCTCGGCGCCGAGTT  TTTCTCTTAGGTTTACCCGC | Functional complementation  Transformants  identification |

**Supplemental Figure 1** Phenotypic characterization of wild type (DJY), *pfp1-1* and *pfp1-2*. **a** DJY, *pfp1-1* and *pfp1-2* plants; **b** Panicles of DJY, *pfp1-1* and *pfp1-2*.


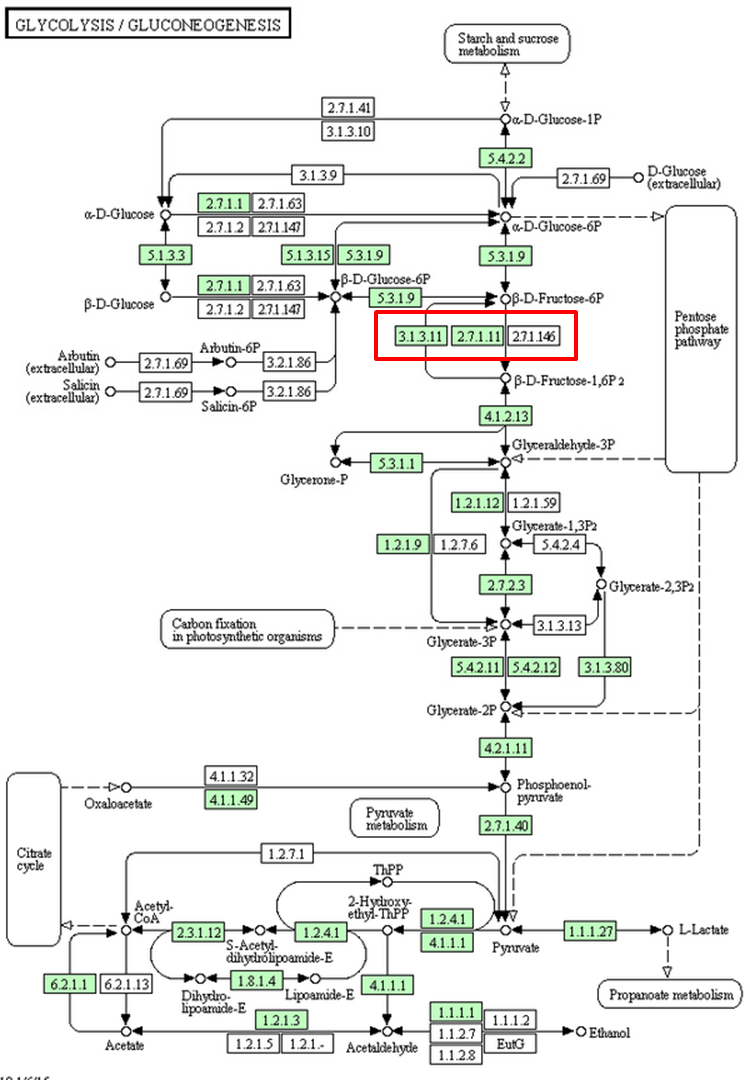


**Supplemental Figure 2** Glycolysis/gluconeogenesis pathway from KEGG. The red box indicates the step where PFP functions.

**Supplemental Figure 3** β-Glucuronidase (GUS) staining of various tissues in the *Pro PFPβ*:GUS transgenic lines. **a** Root; **b** Leaf blade; **c** Leaf sheath; **d** Ligule; **e** Stem; **f** Panicle.


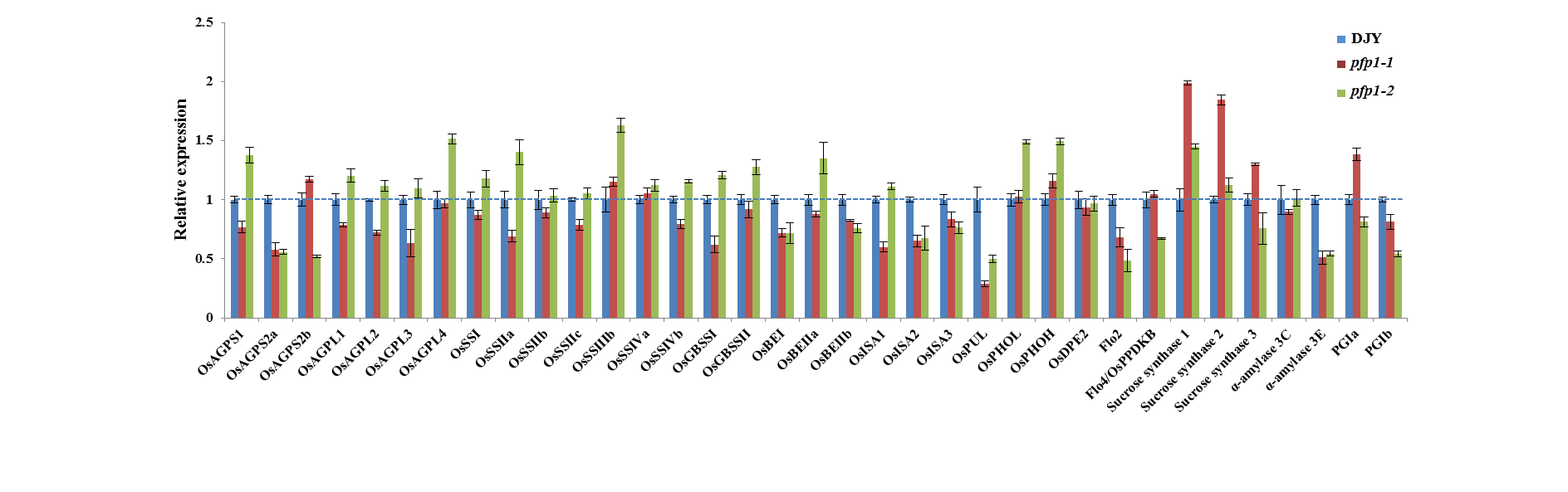


**Supplemental Figure 4** Expression levels of selected starch-synthesis related genes in developing seeds at 12 days post anthesis. Expression levels of selected starch synthesis-related genes in developing seeds at 12 days post anthesis. All data analyzed by qPCR were compared are relative to the wild type (DJY) value set as 1. Values are presented as means ±SD (n=4). AGPS (AGPS1, AGPS2a and AGPS2b) and AGPL (AGPL1, AGPL2, AGPL3 and AGPL4), ADP glucose pyrophosphorylase small subunit and large subunit; SSI, SSIIa, SSIIb, SSIIc, SSIIIb, SSIVa, and SSIVb (soluble starch synthase I, IIa, IIb, IIc, IIIb, IVa, and IVb; GBSSI and GBSSII (granule-bound starch synthase I and II; BEI, BEIIa and BEIIb), starch branching enzyme I, IIa and IIb; ISA1, ISA2, and ISA3, corresponding isoamylase isozymes; PUL, pullulanase; PHOL and PHOH, starch phosphorylase L and H; DPE2, disproportionating enzyme II; Flo2, floury endosperm 2; Flo4/PPDKB, floury endosperm 4/ pyruvate phosphate dikinase B; PGI-a and PGI-b, glucose-6-phosphate isomerase a and b.


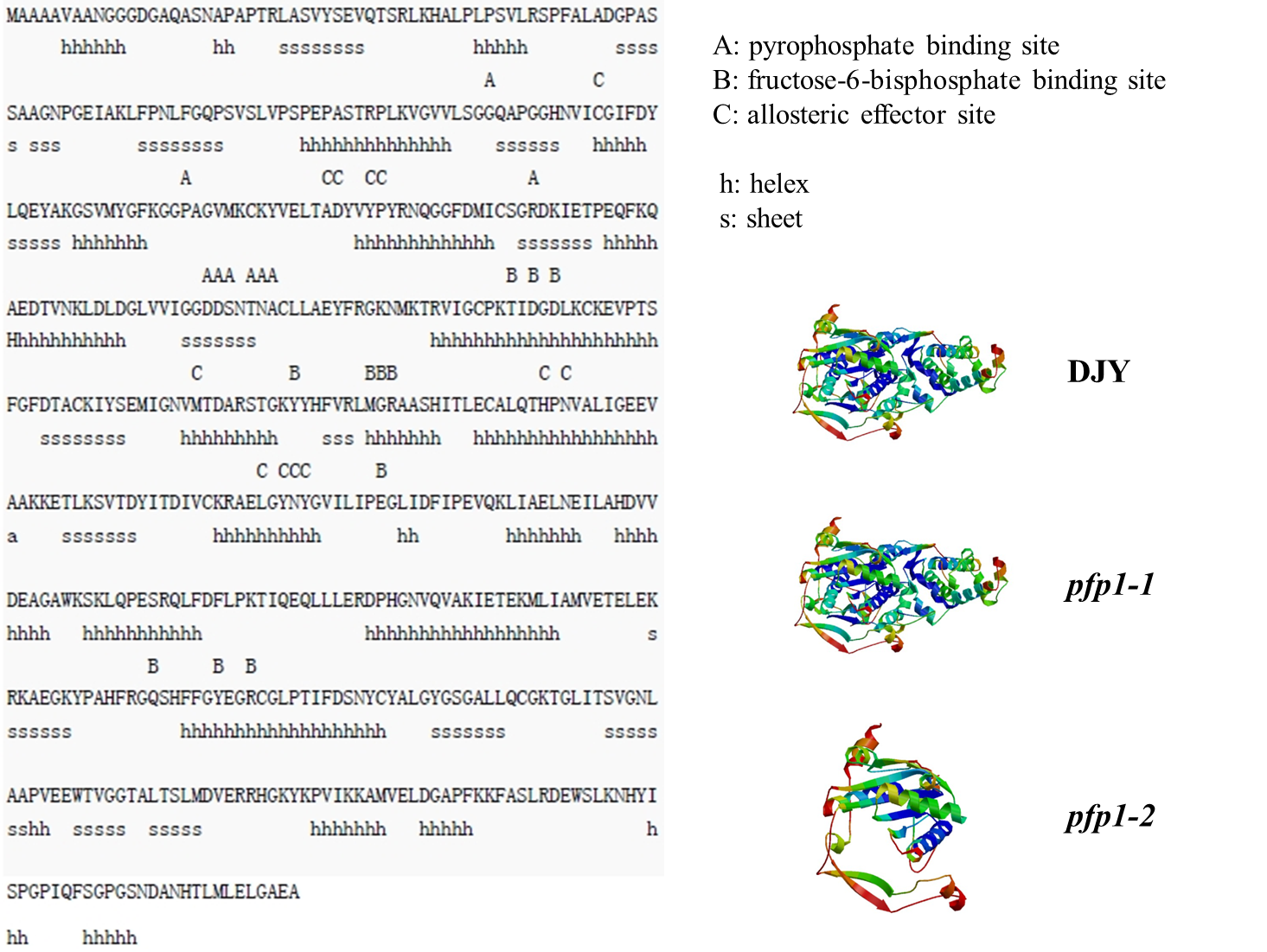


**Supplemental Figure 5** Protein configurations of pyrophosphate: fructose-6-phosphate 1-phosphotransferase (PFPβ) from DJY, *pfp1-1* and *pfp1-2* predicted by the Swiss-Model homology modeling server. Protein configurations of pyrophosphate: fructose-6-phosphate 1-phosphotransferase (PFPβ) from DJY, *pfp1-1* and *pfp1-2* predicted by the Swiss-Model homology modeling server (http://swissmodel.expasy.org/SWISS-MODEL.html). The D to N change in *pfp1-1* did not result in a significant change in configuration, and the reduction in enzyme activity may have been caused by the reduced substrate affinity or catalytic activity. The premature mutation termination of *pfp1-2* changed its basic configuration, almost abolishing the enzyme activity.
